# Evaluation of Strain Sensors Based on Poly(acrylonitrile-*co*-butadiene) and Polypyrrole Synthesized by the Diffusion Method

**DOI:** 10.1021/acsomega.4c02166

**Published:** 2024-05-29

**Authors:** José Carmelo Encinas-Encinas, María Mónica Castillo-Ortega, Teresa del Castillo-Castro, Dora Evelia Rodríguez-Félix, Jesús Manuel Quiroz Castillo, Jesús Alberto Huitrón Gamboa

**Affiliations:** Departamento de Investigación en Polímeros y Materiales, Universidad de Sonora, Hermosillo 83000, Mexico

## Abstract

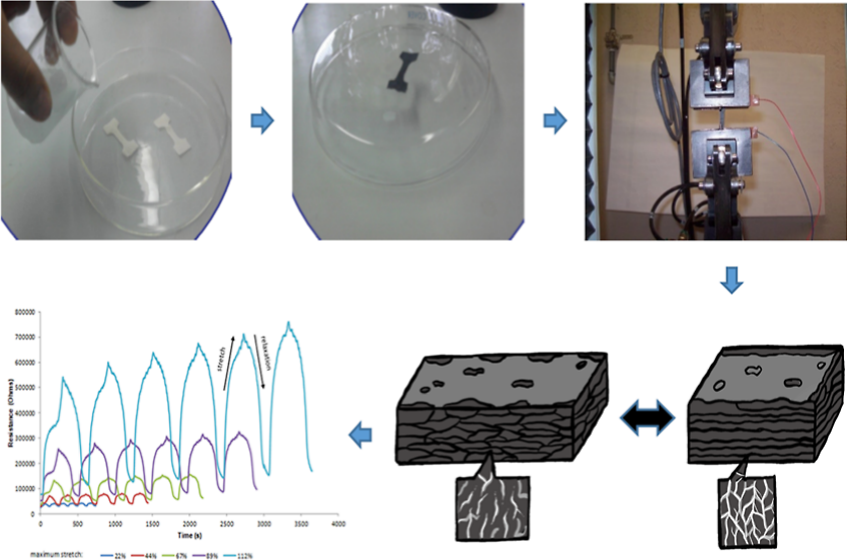

In this study, the functionality of an elastomer composite material containing polypyrrole (PPy) as a stress sensor was evaluated. The material was prepared using the swelling method by diffusing the pyrrole monomer into the elastomer before polymerization. To achieve adequate diffusion, organic solvents with affinity for the elastomer were used. The resulting materials were characterized by scanning electron microscopy (SEM), surface electrical resistance, and thermal and mechanical properties for application as a stress sensor. The simultaneous change in electrical resistance and tension stress was measured using a digital multimeter with electrodes connected to the jaws of a universal mechanical testing machine. The influence of stress cycles on the piezoresistivity of the composite materials was investigated. The obtained PPy/NBR composite presented a good combination of electrical conductivity and mechanical properties. The strain at break remained with mild variation after coating with PPy.

## 1 Introduction

Composite materials that combine electrical conductivity and elastic properties have been intensively studied in recent years.^1,2^ The main interest in these composite materials is their piezoresistivity,^3^ that is, the capacity to change electrical resistance in response to the application of mechanical stress that causes deformation. Piezoresistive materials are useful for manufacturing the sensing elements of sensor devices or indicators of strain,^4^ replacing the traditionally used metal strain gauges. The main advantage of composites over metal strain gauges is their flexibility, which allows for potential applications in coating structures or devices that are implanted in organic environments. Additionally, their application has expanded into robotics^5^ and as interfaces between robots and humans,^6^ as well as in motion detection.^7^

Electrically conductive elastomers (ECEs) are a class of materials that exhibit the piezoresistive effect and have attracted attention based on their mechanical properties, especially high strain and flexibility. The development of ECEs began with the combination of a conductive material with another one, and the latter provided elasticity. Early examples included metal particles dispersed in a highly elastic polymer matrix.^8^ However, high loading percentages, close to 50%, were required to reach the percolation threshold and produce a conductive material, even when using metal nanoparticles with a large dispersion.

Another type of particle used for the preparation of ECEs was carbon black^9^ dispersed in different elastomer matrices. An improvement in the behavior of the ECE was achieved using carbon allotropes such as carbon fibers,^10^ graphene sheets, and carbon nanotubes.^11,12^ Composites with improved mechanical properties were prepared via solution mixing.^13^ Although carbon allotropes have been successful, their costs are relatively high.

An alternative to conducting fillers for ECEs is inherent conducting polymers (ICPs), which have good electrical conductivity and compete with carbon allotropes because of their low cost. ICPs have been incorporated into elastomers using different methods, such as mechanical mixing, in a way similar to the incorporation of carbon black. A porous material consisting of polypyrrole (PPy)/polyurethane was obtained using a prepolymer and solution casting/particle leaching method.^14^ ICPs have the versatility to be blended using two general alternative methods: mixing after polymerization^15^ or mixing the monomer and substrate, followed by in situ emulsion polymerization.^16^ Both of these methods were tested, and promising results were obtained.

The mechanical blending of a polymer solution with toluene as a solvent has been used to evaluate various elastomer/PPy blends.^17^ Silicone rubber/PPy composites were prepared using PPy powder mixed with polymer matrix components by cast molding between two parallel plates.^18^

During the in situ polymerization method, mixing is carried out before polymerization, which allows for a number of changes in the process of incorporating the monomer into the matrix. Some studies report aniline polymerization in ethylene vinyl acetate solution^19^ and solution polymerization of pyrrole in nitrile rubber (NBR) using simultaneous mechanical mixing in a two-roll mill.^20^

PPy-coated nylon fibers were prepared by in situ polymerization of pyrrole mixtures with natural rubber (NR) latex in the presence of nylon fibers.^21^ The conductivity of the NR/PPy composites was enhanced at very high PPy loadings.

Several elastomeric materials have been coated with conductive polymers to obtain flexible and elastic films with electroconductive properties. Pyrrole was electrodeposited onto sheets of NBR/carbon fiber as a current collector to obtain a flexible composite electrode.^22^ Good adhesion was reportedly obtained between the PPy and natural rubber using the vapor phase polymerization technique owing to the diffusion of PPy into the substrate.^23^ The use of organic solvents allowed for a good dispersion to be obtained by the in situ polymerization of PPy/NBR.

Coating materials provided better results than the incorporation of a conductive material into the matrix of the elastomer. To achieve a higher conductivity, there is no need for the addition of conductive components to all the materials, and the elasticity of the original matrix is maintained. Some coatings suffer from low adhesion, depending on the technique used during the application; however, this may be improved if interpenetration is achieved into the matrix surface layer adjacent to the coating.

In recent years, there has been an increased focus on hydrogels as a matrix, either by dispersing conductive particles or providing ionic conductivity.^24^ Hydrogels offer numerous advantages, particularly as biocompatible materials in diagnostic applications that require contact with the human body. However, hydrogels face the disadvantage of dehydration, which alters their elasticity properties. To overcome this issue, we revisited the use of elastomers, which do not suffer from dehydration. A variety of elastomers, such as thermoplastic polyurethane,^25^ silicone rubber,^26^ epoxidized natural rubber,^27^ and carboxylated styrene butadiene rubber,^28^ have recently been employed in the development of stretch sensors.

In our laboratory, we have proposed a new method of in situ synthesis of conductive polymers, which provides high homogeneity. To achieve the interpenetration of the conductive material into the rubber matrix, we synthesized the composite material by diffusing the monomer into the elastomer.^29^ This process involved diffusing a pyrrole monomer into an NBR matrix. In this work, we present an evaluation of the material as a stretch sensor. The objective of this work is to evaluate the piezoresistive response of the fabricated materials and explore improvement options.

## 2 Experimental Section

### 2.1 Sample Preparation

The substrate used to prepare the composite material was NBR sheets with a thickness of 0.07 mm (Ambiderm) that were cut into pieces with dimensions of 30 × 10 mm and washed with distilled water. Pyrrole (Aldrich) previously distilled, acetonitrile, tetrahydrofuran (THF), and copper(II) perchlorate hexahydrate [Cu(ClO_4_)·6H_2_O] were used in this study.

Composite PPy/NBR films were prepared by in situ chemical polymerization of pyrrole using the swelling method with copper(II) perchlorate hexahydrate as the oxidizing agent. NBR sheets were immersed in 6 mL of 0.1 M pyrrole solution in a 4:1 mixture of acetonitrile and THF for 1 min, after which they were removed from the solution and allowed to stand for 1 min. Subsequently, the samples were immersed in 3 mL of a 0.45 M solution of Cu (ClO_4_)·6H_2_O in acetonitrile for 5 min. The samples were removed and dried at room temperature for 24 h. After the samples were dried, they were dipped in acetonitrile to remove the residue from the reaction and were allowed to dry for another 24 h.

### 2.2 Sample Characterization

Mechanical tests were performed using a universal mechanical testing machine (UNITED brand SSTM-5KN model) with a load cell of 5 kN, according to ASTM D 1708-96 for analyzing the microtensile properties of plastics. The tensile strength, elongation, and Young’s modulus of the NBR and PPy/NBR films were measured at 10 mm/min.

The electrical conductivity was analyzed by measuring the volume resistivity of the films using the standard two-point method with a multimeter (STEREN MUL-040). The films were placed between the contact areas of two tungsten electrodes, the electrodes were connected to a multimeter, and the electrical resistance was measured. The surface resistance was also measured by using a multimeter by placing the electrodes on the film surface with a spacing of 1 cm between.

Fourier-transform infrared (FTIR) spectra were recorded in a Frontier spectrometer (PerkinElmer) using the ATR reflection diamond accessory.

For the thermal analysis, the SDT 2960 instrument was used for simultaneous differential scanning calorimetry–thermogravimetric analysis (DSC–TGA) and differential thermogravimetric analysis (DTG). (DTA) Measured samples were measured by weighing approximately 7 mg, which was then heated to 500 °C at a heating rate of 10 °C/min under a nitrogen flow of 24 cm^3^/min.

Photomicrographs were recorded for one of the prepared PPy/NBR films by using a SEM JEOL 5410LV instrument with an electron beam intensity of 15 kV under high vacuum. The sample was washed with acetone, allowed to dry, and stretched until it broke to expose the cross section for observation, and subsequently, the sample was adhered to the metallic specimen holder with carbon tape.

To determine the relationship between strain and the sensor resistance of PPy/NBR, the universal mechanical testing machine was used to stretch the sample at a controlled rate while simultaneously measuring the electrical resistance of the sensor using a digital multimeter (Agilent model 34410) with two copper electrodes connected to the ends of the PPy/NBR film. The multimeter was connected to a computer for data capturing.

The gauge factor, a measure of the sensitivity of a strain or stress sensor, was utilized to study the piezoresistive behavior. Defined as the relative change in the electrical resistance of a sensor material divided by the amount of mechanical deformation experienced by that material, the gauge factor indicates how much the electrical resistance of a material changes in response to a certain amount of mechanical deformation. It is calculated using the formula *G* = (Δ*R*/*R*_0_)/ε, where Δ*R*/*R*_0_ represents the relative change in resistance and ε denotes the applied strain.^30^

## 3 Results and Discussion

### 3.1 Mechanical Properties

The mechanical properties of NBR and PPy/NBR are listed in Table 1. According to the results, the tensile strength and Young’s modulus of the composite material decreased with the incorporation of PPy. In this case, a plasticizing effect was observed. Furthermore, the elongation at rupture did not change significantly. In previous studies, it was found that the addition of conductive polymers to various substrates served as a reinforcing filler, which increased the Young’s modulus. However, a significant decrease in the elongation at rupture highlights the limitation of the practical application of these materials. In this study, a high breaking strain value for deformation owing to tensile stress is favorable for application of the composite material as a sensor. Previous studies have been conducted using blends of various elastomers with a conductive polymer; in most cases, either the mechanical or electrical properties of the material are too weak to achieve the objectives. Unlike the aforementioned polymer blends, the PPy/NBR films synthesized in this study have a good combination of electrical conductivity and mechanical properties.

**Table 1:** Mechanical Properties of the NBR and PPy/NBR

	ultimate strength (MPa)	strain fracture (%)	Young’s modulus (MPa)
NBR	19.1 ± 3.1	460 ± 36	9.3 ± 3.5
PPy/NBR	6.6 ± 1.1	459 ± 35	6.6 ± 1.3

The mechanical properties are provided by the elastomer (NBR) as the continuous phase. On the other hand, PPy is characterized by its brittle nature and low resistance to tensile stress. When blends of these polymers are not homogeneously performed, the PPy phases tend to weaken the material. Therefore, dispersed PPy phase sizes as small as possible are required to achieve a material with properties similar to those of the original elastomer. Additionally, the theory of conductivity in composite materials is based on the percolation theory. To increase the electrical conductivity of the material, reaching the percolation threshold is necessary, where the conducting particles are in contact with each other and there are no voids that isolate the conductivity.

### 3.2 Infrared Spectroscopy (FTIR)

**Figure 1:**
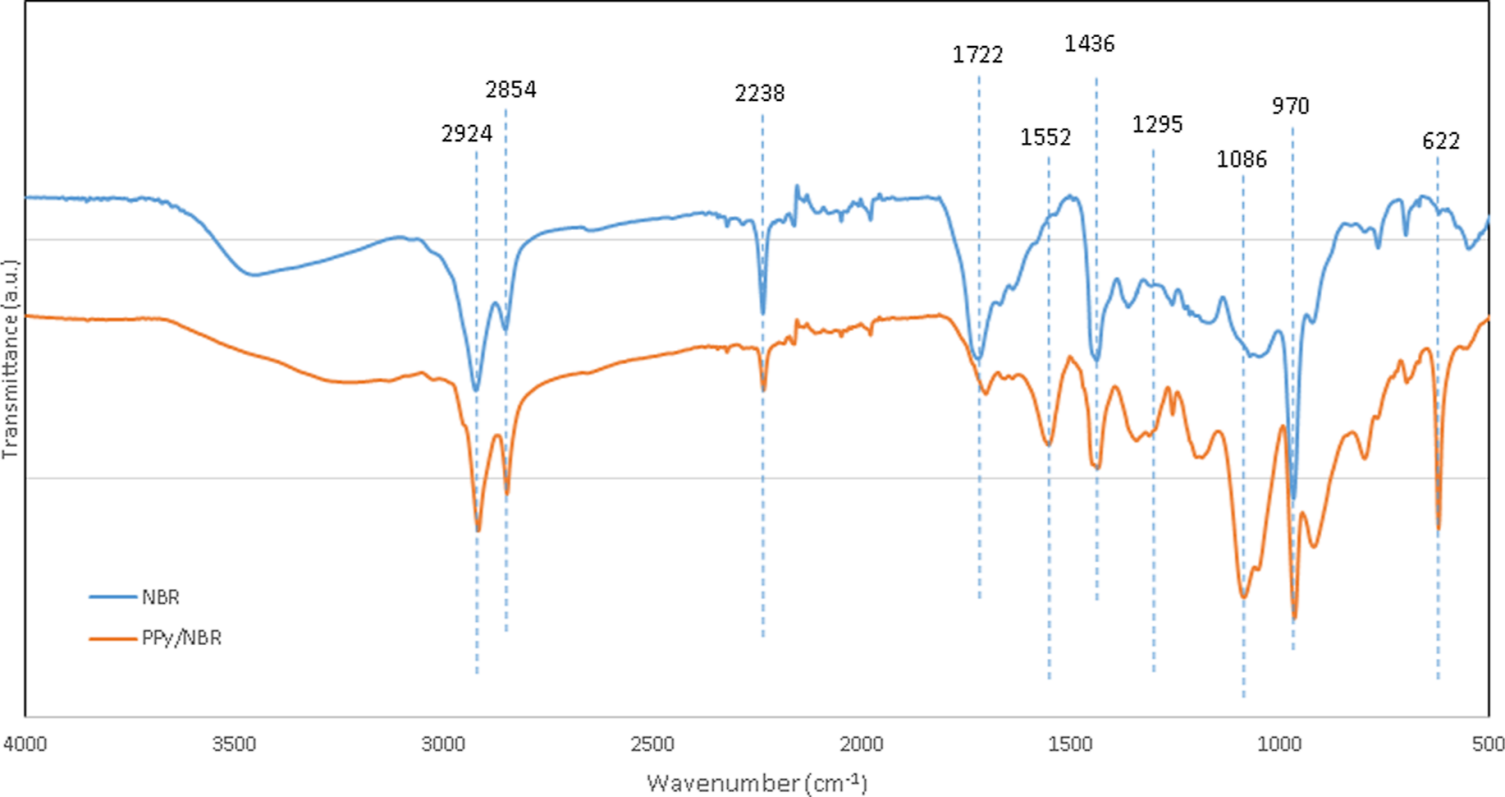
FTIR spectra of NBR and PPy/NBR.

Figure 1 shows the FTIR spectra of NBR films and PPy-coated NBR films.

The spectrum shows the characteristic bands of NBR: two peaks at 2924 and 2854 cm^–1^ associated with the C–H stretching vibration, the 2238 cm^–1^ peak corresponding to the stretching vibration of the carbon–nitrogen triple bond of the nitrile group. A strong absorption band at 1722 cm^–1^ associated with the stretching vibration of C=O, the signal at 1436 cm^–1^ attributed to the C–H bending vibration in CH_2_, and the band at 970 cm^–1^ related to the out-of-plane bending vibration of C–H.^31−33^

After coating, the characteristic bands of PPy appear: a strong absorption band at 1552 cm^–1^ associated with the stretching vibration of the conjugated carbon–carbon double bonds (C–C/C=C) in the PPy ring, and the bands at 1295 cm^–1^ attributed to the =C–H in plane vibration. The band at 1086 cm^–1^ assigned to =C–H in plane deformation vibration, and the peak at 622 cm^–1^ associated with C–H wagging.^34,35^ Others bands of PPy are overlapped by the absorption bands of NBR.

### 3.3 Thermal Analysis

**Figure 2:**
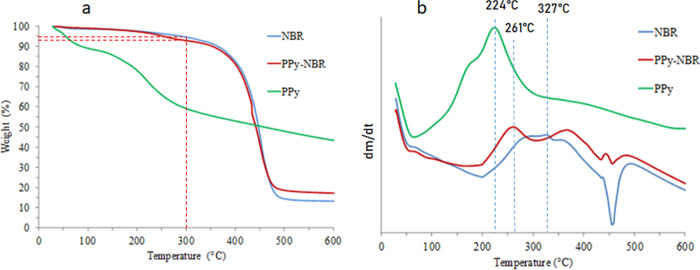
Thermograms of pure and composite components: (a) TGA and (b) DTG.

The thermal behavior of the composites is shown in Figure 2a, and the PPy has a lower thermal stability than the NBR and exhibited a mass loss of 10% at temperatures lower than 50 °C. This is attributed to the evaporation of residual solvents remaining after the synthesis process. A second mass loss was observed at 150 °C, which is attributed to the loss of the remaining oxidizing agent, and a third mass loss at 200 °C is attributed to the degradation of PPy chains. The NBR sample exhibited a single mass loss at 400 °C, while the mixture of PPy and NBR exhibited a combination of both compounds, with a slight deviation at a temperature of 300 °C associated with the degradation of PPy and the composite material. The TGA curves for the PPy/NBR and NBR samples are similar, with only a small deviation of approximately 2% around the temperature of 300 °C. This slight deviation is due to the presence of PPy in the mixture. The similarity between the PPy/NBR curves and the NBR curve is almost exact, owing to the low amount of PPy in the mixture. Although the quantities of PPy in the material are low due to the synthesis process, they are sufficient to achieve adequate electrical conductivity for use as a strain sensor.

Materials with PPy have a greater thermal resistance and maintain their mass up to 250 °C. The presence of PPy between the NBR chains provided enhanced interactions between these components and improved their resistance to the temperature. It is frequently reported that an increase in the amount of PPy in the composite mixture reduces the decomposition initiation temperature,^21^ and the weight loss is lower at temperatures above 450 °C.^36^

The DTG curves in Figure 2b show an enhancement in the maximum decomposition temperature of the composite material; the maximum exotherm observed for PPy was 224 °C, whereas that of NBR was 327 °C, and that of the composite material was observed at 261 °C. The displacement of the decomposition initiation temperature was significant for the composite material, which indicates an effective dispersion of PPY throughout the NBR film owing to the diffusion of pyrrole in the NBR prior to polymerization. Thermal stability is related to the dispersion of particles.^37^

### 3.4 Scanning Electron Microscopy

**Figure 3:**
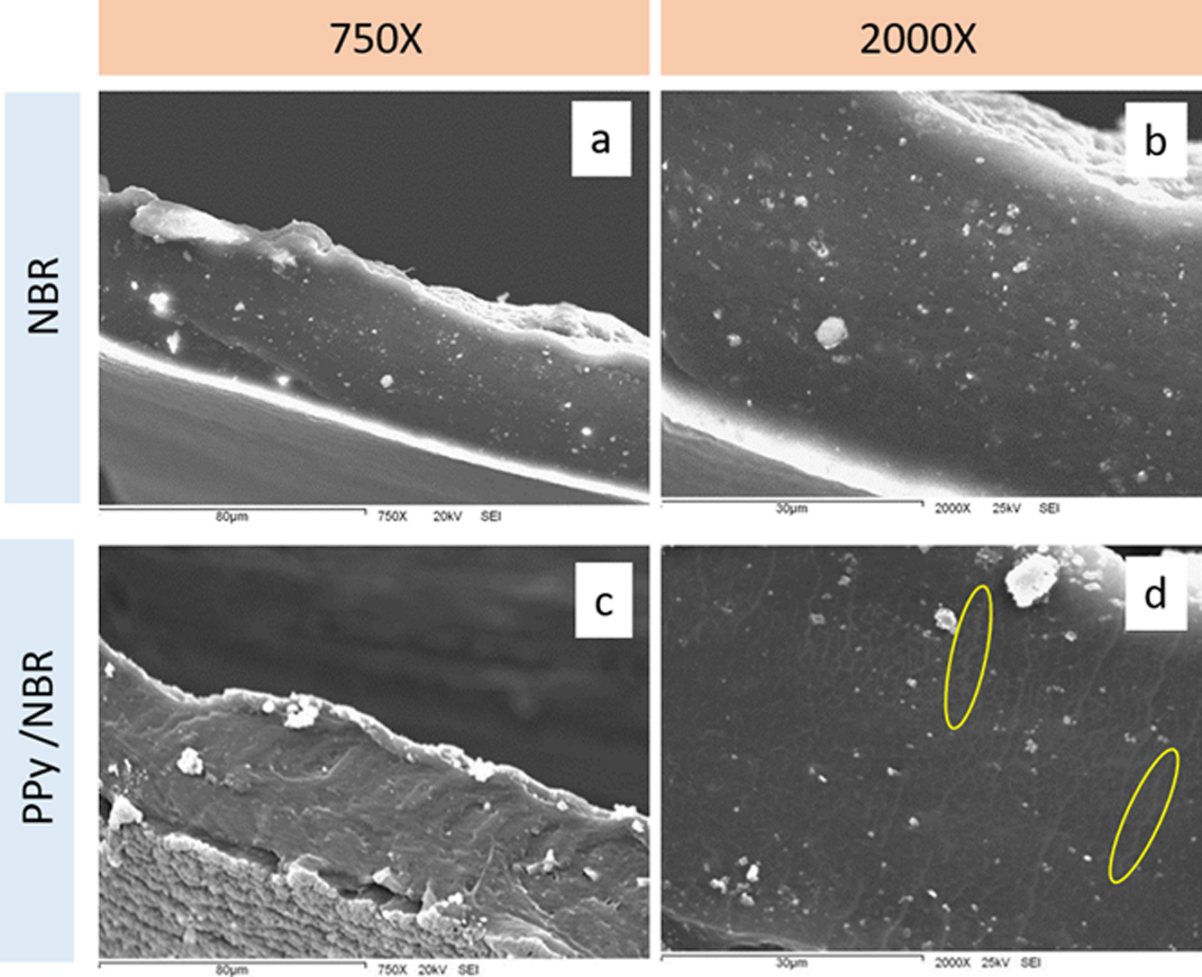
SEM micrographs of cross sections of NBR (a,b) and PPy/NBR (c,d) at 750× (a,c) and 2000× (b,d) magnifications.

The micrograph images presented in Figure 3 depict various characteristics of NBR samples both in their pure state and in the presence of PPy (PPy/NBR). In Figure 3a, at 750×, a portion of the lateral surface of pure NBR is observed, which exhibits a smooth texture, while the cross section shows a dense and uniform material. At magnification to 2000× in Figure 3b, a dense material without cracks is distinguished, accompanied by some white spots reflecting the ductile fracture of the elastomer.

In Figure 3c, a layer of PPy forms on the surface of the NBR film, while the cross section continues to maintain a uniform hue. This suggests a heterogeneous distribution of PPy in the sample, and Figure 3d shows transverse lines indicated with a yellow oval. These lines, branching from the surface toward the central part of the film, evidence the penetration of the conductive PPy into the NBR. This phenomenon favors percolation, crucial for imparting electrical conductivity in the composite material.

### 3.5 Piezoresistivity Behavior

The electrical conductivity of PPy/NBR was 1 × 10^–4^ S/cm, a higher value compared to that of pristine NBR. This suggested a more homogeneous distribution of PPy in the NBR matrix.

**Figure 4:**
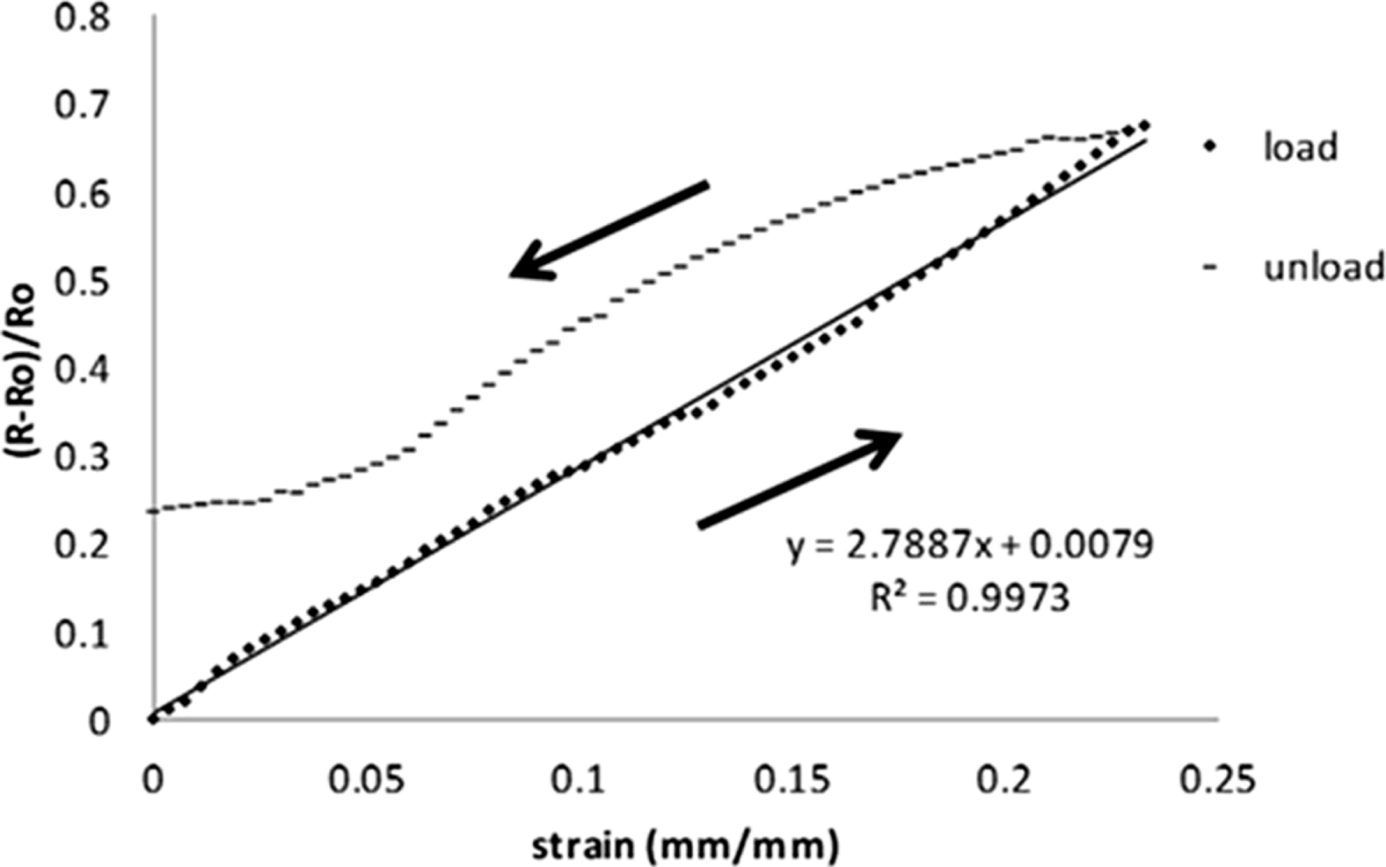
First cycle of normalized electrical resistance plotted against applied strain in the elastic region for the composite PPy/NBR.

To determine the piezoresistivity response of the PPy/NBR composite, the films were subjected to cyclic loading of uniaxial tensile strain of up to 22%. Figure 4 shows the tensile strain and relative resistance change [(*R* – *R*_0_)/*R*_0_] response, where *R*_0_ is the initial value of the electrical resistance, and *R* is the instantaneous resistance.

The change in the electrical resistance of the PPy/NBR composite upon stretching causes a strain of up to 0.22. The graph of the electrical resistance change versus strain can be fitted to a straight line, starting from the origin with a slope of 2.7887. The linearity of the graph indicates that the PPy/NBR composite is a suitable material for application in a stress-sensor device. Figure 3 also shows the values of the change in the electrical resistance when the charge is released and the composite returns to its original dimensions. The resistance change values deviated from the initial stretch values, which was caused by the delay in returning to their original dimensions. A longer time is required to allow the dispersed PPy particles in the matrix to form new pathways for conduction and decrease its resistivity. When returning to its original dimensions, a nonzero electrical resistance change value remained for the PPy/NBR composite at 0.25 (*R* – *R*_0_)/*R*_0_, as shown in Figure 4.

The deviation in resistivity observed at the end of the measurement may affect the operation of the device to measure deformations; therefore, a sequence of stretching was performed, and the response was plotted as the change in electrical resistance.

**Figure 5:**
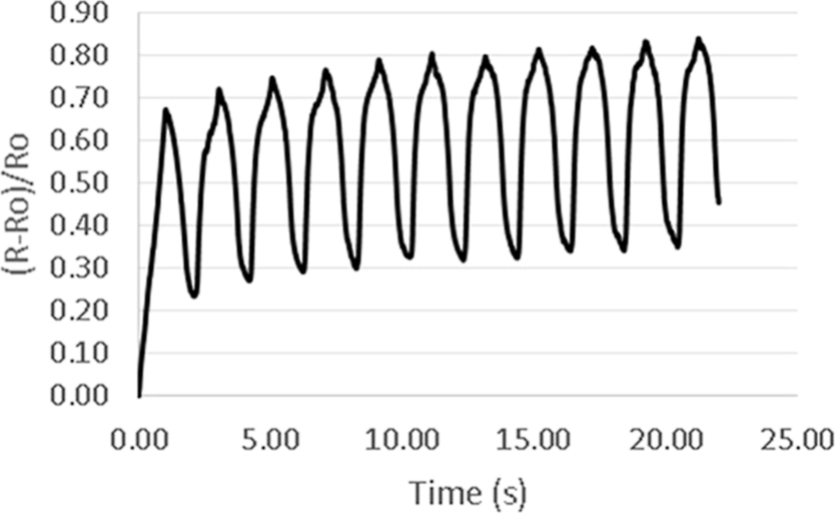
Normalized electrical resistance vs time during 11 cycles of stretching.

In Figure 5, the electrical resistance response of the material was measured by applying a sequence of 11 cycles of stretching deformation and contraction, with the maximum elongation measured at 22% strain. The increase in the maximum electrical resistance after each cycle is clearly visible in Figure 5; subsequently, the maximum electrical resistance stabilized after the sixth cycle. The increase in electrical resistance is attributed to the separation of conductive particles during each stretching cycle, which reached a limit after the sixth cycle and stabilized the electrical conductivity.

After the first charge and discharge cycle, the shape of the line changed from a straight line to a curve that changed the slope after normalized resistance values reached close to 0.6.

**Figure 6:**
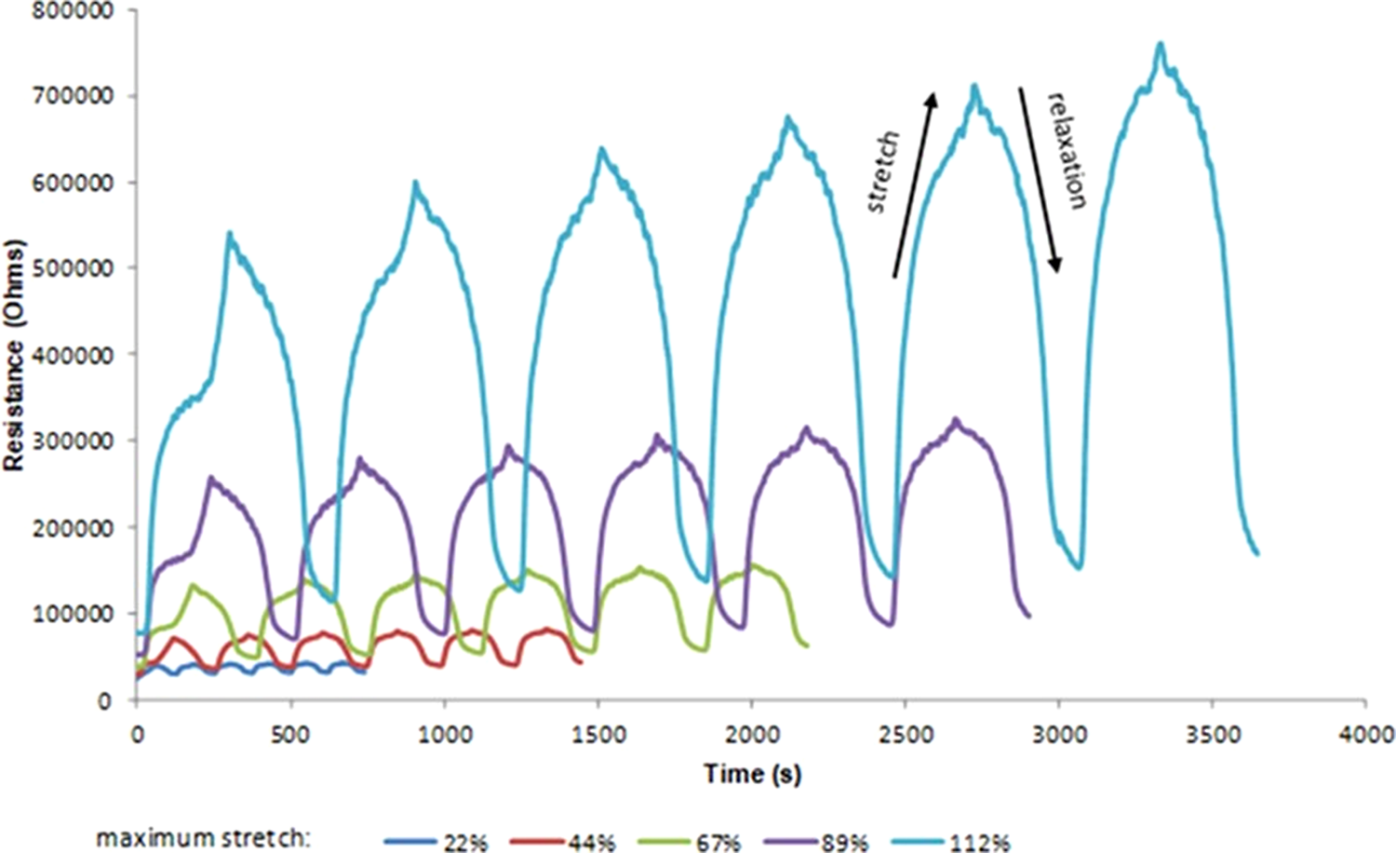
Electrical resistance vs time during 6 cycles of stretching.

As shown in Figure 6, as more cycles are performed and/or greater stretching is applied to the samples, the signal exhibited sinusoidal behavior. This is important because, by performing an electronic interpretation or handling the sensor, fewer circuits will be required for its operation. As the tests were conducted with a greater stretch length, the resistive values increased in an acceptable range; 38.4 kΩ for test cycle 1 at 22% strain, 802.1 kΩ for cycle 8 at 112% strain, and handling occurred in intervals of expected values, that is, without reaching values greater than 200 MΩ.

During the process of relaxation of the sample for each cycle, a recovery was achieved close to the initial conditions of the sample; that is, the elastic properties of the sample were retained to a good degree, without exhibiting plastic behavior.

The increase in the minimum and maximum resistance values during each cycle may be attributed to the Mullins effect, which considers that in each cycle, a greater applied force is required, caused by a loss of stiffness in the sample.^38^

The recovery of the electrical resistance was close to the initial values after each cycle, which may be related to the maintenance of elastic properties without plastic behavior.

To calculate the gauge factor, only the stretching phase was plotted for each cycle and overlapped by setting the origin of the deformation to zero. The slope of the initial part of the curve fits the slope that is characteristic of each series very well, and the values are listed in Table 2. The slope of the final part of the curve had greater variation, with a tendency to increase after each cycle. Zetina-Hernández et al.^12^ attributed this result to elastic and plastic deformations. The point of change between the two slopes nearly corresponds to the yield point of the material at approximately 0.1 strain (nominal strain).^38^

**Table 2:** Gauge Factor for Different Stretching Tests

strain %	final length (mm)	initial length (mm)	gauge factor 1	gauge factor 2
22	27.3	22.3	8.89	0.95
44	32.3	22.3	13.3	1.29
67	37.3	22.3	20.72	1.60
89	42.3	22.3	25.32	2.44
112	47.3	22.3	35.37	3.94

**Figure 7:**
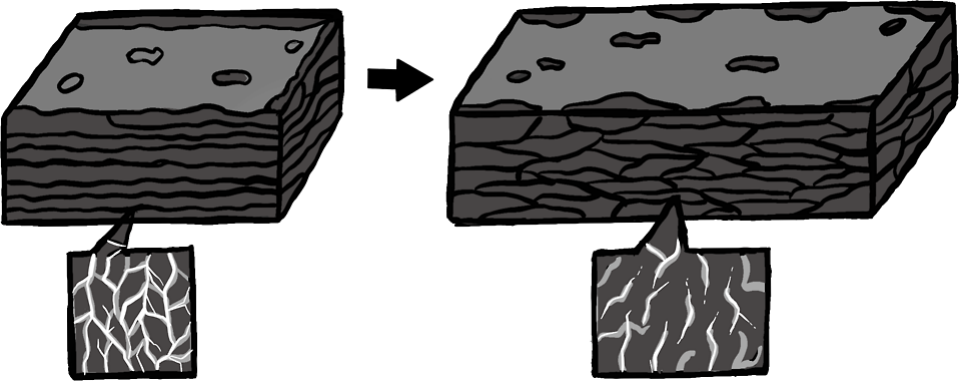
Schematic drawing of percolation in PPy/NBR films when deformation is applied.

The percolation model governs the electrical conductivity of the material. It involves conductive particles embedded within the rubber, which serve to protect the conductive path. When the rubber undergoes deformation, cracks form, interrupting the flow of conductivity and resulting in increased resistivity (Figure 7). However, once the external force is removed, the rubber reverts to its original shape, sealing the cracks and enhancing the contact between the conductive particles. As a result, the material recovered its conductivity.

In contrast, materials that rely solely on a conductive coating for recovery experience a reduction in crack formation, but the contact between particles does not fully restore the original state. Consequently, residual empty spaces remain, preventing the complete recovery of electrical conductivity.

To address this issue, PPy is synthesized by using the swelling method, which creates conduction paths within the inner portion of the rubber. When the rubber is deformed, the connections between the conductive particles are reduced, leading to a decrease in conductivity. However, during the recovery process, the enveloping forces exerted by the rubber result in improved contact between the particles. This enhanced contact is achieved due to the rubber’s surrounding action, allowing for better recovery compared to surface-coated materials. Consequently, the resistivity of the material is less affected when compared to that of materials that solely rely on a coating.

## 4 Conclusions

In this study, a semiconductor-based material in an NBR matrix was developed by using the swelling method with a conductive polymer as a filler material. The resulting composite material demonstrated a comparable elongation at break value to that of the NBR elastomer alone, suggesting its suitability for use as a deformation sensor due to its high elongation at break value. Thermal analyses revealed a low PPy content in the sample, approximately 2%, which had a minimal influence on the elongation values of the composite material.

The piezoresistive behavior of PPy/NBR composite films under cyclic loading of uniaxial tensile strain was investigated. Initially, a linear relationship between the electrical resistance change and strain was observed, indicating the potential application of the composite in stress-sensing devices. However, deviations in resistivity were noted, potentially impacting the deformation measurements. Despite this, subsequent cycles of stretching and relaxation showcased sinusoidal behavior in electrical resistance. The samples exhibited resilient elastic properties after each cycle with minimal plastic behavior, albeit with an increase in resistance values attributed to the Mullins effect. Gauge factor calculations underscored the influence of elastic and plastic deformations, with a discernible change in slope near the material’s yield point. In summary, these findings contribute to a deeper understanding of the piezoresistive response of PPy/NBR composites, offering insights into the development of stress-sensing devices with enhanced reliability.
